# 

**Published:** 1855-03

**Authors:** 


### IOWA
### MEDICAL JOURNAL.

CONDUCTED BY THE FACULTY OF THE

MEDICAL DEPARTMENT OF THE IOWA UNIVERSITY.

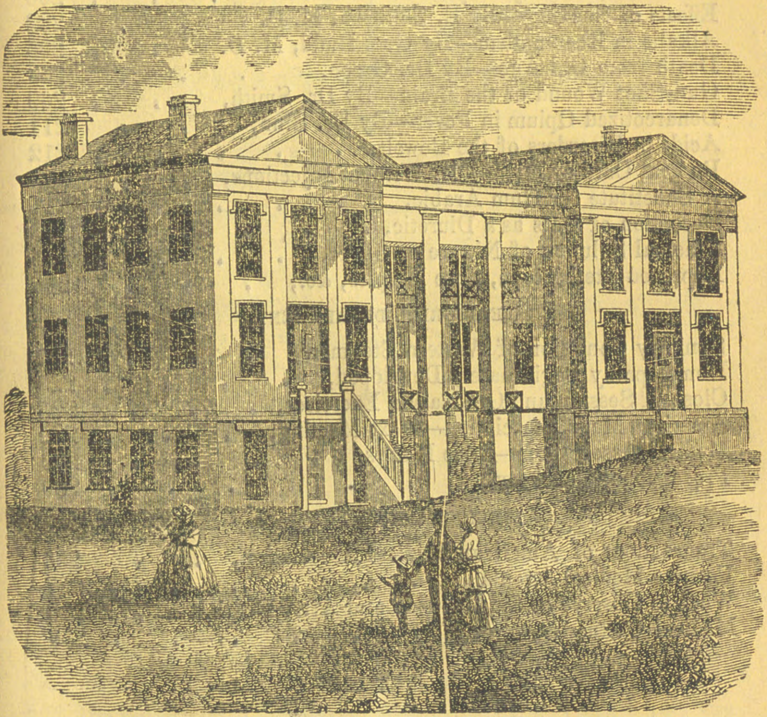

PUBLISHED EVERY ALTERNATE MONTH.

TERMS—TWO DOLLARS, IN ADVANCE.

KEOKUK, IOWA:

PRINTED AT THE WHIG BOOK AND JOB OFFICE.

☞ Address all Communications, post-paid, to "Medical College, Box 109, Keokuk, Iowa. ☜

